# Pre-eclampsia and barker’s hypothesis: are we beginning to see the trees within the forest?

**DOI:** 10.1038/s41390-024-03264-7

**Published:** 2024-05-31

**Authors:** Stephanie M. Tsoi, Martina Steurer, Emin Maltepe, Jeffrey R. Fineman

**Affiliations:** 1 Department of Pediatrics, University of California San Francisco, San Francisco, CA USA; 2 Department of Epidemiology and Biostatistics, University of California San Francisco, San Francisco, CA USA; 3 Cardiovascular Research Institute, University of California San Francisco, San Francisco, CA USA

Dating back from Barker’s landmark reports beginning in 1986, abnormalities in the intrauterine environment have been suspected to influence the development of diseases later in life. Now referred to as Barker’s hypothesis, abnormalities in pregnancy have been associated with several childhood and adult diseases including systemic vascular, pulmonary vascular, and coronary artery diseases. The cellular and molecular explanations for Barker’s hypothesis have been postulated to be rooted in epigenetics and altered tissue differentiation throughout the life stages.^1^ In the current issue of *Pediatric Res*, Kua et al.’s study provides novel insight into an early phenotype and unique biomarkers that may elucidate Barker’s hypothesis using the specific example of an abnormal intrauterine environment from pre-eclampsia (Pre-E) and its influence on the development of abnormal vascular reactivity.

Pre-E, a significant source of maternal and neonatal morbidities, occurs in up to 10% of pregnancies. Although the comprehensive etiology is not definitive, Pre-E is thought to, at least in part, be due to an abnormal development of the placental vasculature that results in a low flow, high resistance utero-placental unit. Immune dysregulation and inflammation are also important contributors.^2^ This abnormal unit induces many aberrations that include a hypoxic-ischemic intrauterine environment and fetal growth restriction, along with the release of antiangiogenic, vasoconstricting, and inflammation promoting factors. These pathologies are then responsible for setting the stage for both offspring and maternal disorders. Offspring pathology spans multiple systems - CNS, respiratory, gastrointestinal, renal, immunologic, and cardiovascular (including vascular endothelial dysfunction and subsequent vascular disease). Maternal pathology, often referred to as maternal-placental syndromes, includes an increased risk of cardiovascular disease.^3^

The thoughtfully designed study by Kua et al. utilizes non-invasive tools to compare both vascular structure and function in 6-month-old healthy controls and infants born to mothers with Pre-E. In addition, blood was sampled to compare 17 serum biomarkers between the groups. They found no difference in systemic blood pressure or microvessel density, but there was a difference in vascular reactivity in their adjusted model. Kua et al. also showed an increase in two cytokines – IL-8 (a pro-inflammatory marker) and Angiopoeitin-2 (a growth factor involved in angiogenesis and vascular remodeling) in infants born to Pre E mothers. Most interesting was that they found a negative association between IL-8 and vascular reactivity. Kua et al. expertly demonstrated that the childhood and adult-onset vascular diseases associated with Pre-E likely emerge during infancy.

Kua et al.’s findings are novel and add to our limited understanding of the complex interplay between abnormal placentas, fetal inflammation, and post-birth outcomes. Kua et al. has elegantly chosen outcomes that measure both the structure, development, and function of the highly susceptible vasculature. However, some limitations and inconsistencies with previous studies are noteworthy. For example, in the current study non-invasive systemic blood pressure was not different between the groups. This contradicts a previous investigation that demonstrated increases in systolic, diastolic, and mean systemic blood pressures in the offspring of Pre-E mothers from day 2 to 4 weeks of life.^4^ In this study, Chourdakis et al. only investigated offspring from early onset Pre-E (<34 weeks gestation). This distribution of early vs. late onset Pre-E is not documented by Kua et al., but separate analysis was likely not possible given the sample size limitations of the current study. Like many pathologies, pregnancies associated with Pre-E have similarities as well as differences that would be important to delineate when attempting to discern mechanisms of disease. From the contradictory results with blood pressure, it appears that the timing of Pre-E during gestation is critical. In fact, differences in several growth factors have been documented between early and late onset Pre-E.^5^ Additionally, larger cohort studies could control for several vital maternal and pregnancy factors including severity of Pre-E, antenatal steroid use, maternal BMI, and mode of conception. For example, in-vitro fertilization (IVF) is associated with a high incidence of Pre-E, placental pathology, pre-term delivery, and childhood and adult-onset vascular disease which may have overlapping, as well as independent, pathologic mechanisms.^6^ In fact, differences in these variables, including IVF use across groups, may have confounded the findings in the current study. Importantly, it is unfortunate that gestational age at birth could not be adequately matched between the groups (Kua et al. Table 1, >1 week gestation older in controls). Although adjusted for in their models, gestational length is such a strong confounder that direct matching would be ideal and facilitate the ability to better adjust for other variables. Finally, the lack of correlation with placental pathology is a lost opportunity to leverage the placenta to advance neonatal care.^7^ The presence and degree of maternal vascular malperfusion and/or histologic evidence of inflammation, for example, can be highly informative with respect to underlying etiology as well as long term disease susceptibility.^8^

Kua et al. should be applauded for not only assessing both vascular structure and function, but doing so in a non-invasive manner that facilitated study enrollment in a vulnerable patient age group. This allowed studies at an age interval (6 months) that had not been previously investigated. However, with these non-invasive assessments come inherent shortcomings. For example, as the authors nicely discuss, the functional assessment of vascular structure utilizing microvascular density could be improved with newer dark field microscopy and the addition of re-perfusion measurements. In addition, the assessment of vascular reactivity was performed utilizing acetylcholine (Ach) dose responses. Ach is a classic endothelium-dependent vasodilator that facilitates nitric oxide (NO) release following Ach receptor binding. More commonly, and more uncomfortable, flow mediated vasodilation is utilized, which also assesses NO-induced vasodilation using the physiologic flow (shear stress) stimulus, bypassing the need for any receptor binding. Although blunted Ach responses almost certainly reflect impaired NO release secondary to endothelial dysfunction, potential aberrations in Ach receptor density and/or affinity cannot be excluded. In addition, often the assessment of endothelium-independent vasodilation is also tested with NO-donors, such as nitroprusside, that can assess aberrations in vasodilation beyond endothelial function. Although the addition of these assessments would add value, the approach that Kua et al. undertook is completely understandable given the young patient population. However, we cannot lose site of the fact that the diagnostic and prognostic value of these methodologies requires further investigation.

To begin to explore the potential inflammatory-induced changes, Kua et al. performed a venipuncture to determine and compare cytokine profiles. Importantly, their assessment discovered both similarities and differences in cytokine profiles. In a secondary analysis they also demonstrate an inverse relationship between IL-8 levels and vascular reactivity. This fascinating data may shed light on not only a potential biomarker of future disease, but also provide insight into potentials mechanisms of pathology, since IL-8 has been implicated in both endothelial and smooth muscle cell proliferation.^9^ In fact, continued assessment of these profiles with increasing age could provide important mechanistic insights.

We thank Kua et al. for contributing these valuable results to our greater understanding of the long-lasting impact of the intrauterine environment on the outcomes of offspring. This area of research will greatly benefit from further scientific inquiry, and we hope that this study’s findings will prompt future research that aims to better understand Barker’s hypothesis on a cellular, molecular, and biochemical level. This includes the need for large-scale multi-institutional prospective studies that involves careful pathologic examination of the placenta, maternal, umbilical, and sequential postnatal blood sampling, and sequential assessments of cardiovascular structure and function. Only then will we begin to see the trees within the forest of the Barker hypothesis, that could lead to important mechanistic insight facilitating novel treatment and prevention strategy discovery.
